# Prevalence and risk factors of anemia in internationally adopted children: a cohort study

**DOI:** 10.1186/s13052-025-01944-6

**Published:** 2025-03-25

**Authors:** Danilo Buonsenso, Ugo Maria Pierucci, Anna Camporesi, Francesca Raffaelli, Maria Chiara Barbieri, Lucia Scarlato, Arianna Turriziani Colonna, Mariella Iademarco, Ilaria Lazzareschi, Piero Valentini, Gruppo di Lavoro Nazionale del Bambino Migrante della Società Italiana di Pediatria

**Affiliations:** 1 Department of Woman and Child Health and Public Health, Fondazione Policlinico Universitario A Gemelli IRCCS, Largo A. Gemelli 8, Rome, 00168 Italy; 2 Area Pediatrica, Dipartimento Di Scienza Della Vita E Sanità Pubblica, Università Cattolica del Sacro Cuore, Rome, Italia; 3 Department of Pediatric Surgery, “Vittore Buzzi” Children’s Hospital, Milano, Italy; 4 Department of Pediatric Anesthesia and Intensive Care Unit, “Vittore Buzzi” Children’S Hospital, Milan, Italy; 5 Dipartimento Di Scienze Mediche E Chirurgiche, UOC Di Malattie Infettive, Fondazione Policlinico Universitario A. Gemelli IRCCS, Rome, Italy

## Abstract

**Background:**

Adoptive children, who have often experienced inadequate healthcare, malnutrition, and exposure to infectious diseases in their countries of origin, are vulnerable to a range of health problems among which anemia is a major one, potentially leading to long term sequelae. We aimed to investigate the prevalence and risk factors associated with anemia in a cohort of internationally adopted children evaluated at the Pediatric Clinic of the Policlinico Universitario “A. Gemelli” in Rome. between 2007 and 2023.

**Methods:**

Retrospective cohort analysis. Demographic and hematological data were collected for each child. Associations between categorical variables was studied with Pearson’s or Fisher’s test and between quantitative and qualitative variables with Analysis of Variance (ANOVA) with Bonferroni correction. The occurrence of a Hb level inferior to -2SD and that of presenting together Hb Z score < -2SD for and low ferritin have been considered as a binary outcome and studied with multivariable logistic regression models.

**Results:**

Nine hundred and sixty-nine children have been enrolled. Weight and height were significantly lower in children from Asia and India compared to Africa and Latin America. Hb z-scores were significantly lower in the 11–18 age group compared to all other age groups. In univariate analysis, Hb z-scores were associated with black skin color and the presence of parasites in stool. Hemoglobin levels were not associated with patient BMI, creatinine levels, bilirubin, TSH, FT3, FT4, AST, or ALT. The mean corpuscular volume (MCV) was associated in univariate analysis with age at arrival, skin color, Macro-area of origin, duration of institutional stay, iron levels. At same MCV, Hb was higher in Asia compared to Africa (*p* < 0.001). 55 patients had both Hb and MCV values below two SD. These patients are predominantly characterized by black skin color and originating from Africa and India.

**Conclusions:**

There is possibly a complex interplay between environmental exposures and genetic predispositions in shaping the health outcomes of adopted children. Healthcare providers who care for internationally adopted children should prioritize comprehensive health assessments that include screening for anemia, nutritional deficiencies, and parasitic infections.

**Supplementary Information:**

The online version contains supplementary material available at 10.1186/s13052-025-01944-6.

## Introduction

International adoption involves a complex and often challenging process that brings children from diverse socio-economic and geographical backgrounds into new environments. These children, who have often experienced inadequate healthcare, malnutrition, and exposure to infectious diseases in their countries of origin, are particularly vulnerable to a range of health problems upon their arrival in their adoptive countries [1, 2]. Among the most pressing concerns is anemia, a condition characterized by reduced hemoglobin levels that can have far-reaching consequences on a child's physical and cognitive development [3].

The causes of anemia in internationally adopted children are multifactorial, often stemming from a combination of nutritional deficiencies, infectious diseases, and genetic factors [4]. The assessment and management of anemia in this population are further complicated by the lack of reliable health documentation and the varying healthcare standards in the children’s countries of origin [5]. Understanding the prevalence and risk factors associated with anemia in this group is essential for developing targeted interventions that can improve their health outcomes and overall quality of life.

This study was conducted with the aim of assessing the prevalence of anemia and identifying key demographic and health-related factors that influence hemoglobin levels in a cohort of internationally adopted children evaluated at the Pediatric Clinic of the Policlinico Universitario “A. Gemelli” in Rome. By identifying these factors, we hope to provide healthcare providers with the necessary insights to implement interventions that are tailored to the unique needs of this population.

## Materials and methods

The study was designed as a retrospective cohort analysis, including 969 children who were adopted internationally between 2007 and 2023. All the children were evaluated at the Pediatric Clinic of the Policlinico Universitario “A. Gemelli” in Rome, Italy. The data for this study were collected from the medical records maintained at the clinic.

The demographic data collected for each child included their age at the time of adoption, sex, skin color (using a simplified version of the Von Luschan scale [6]), country of origin, and latitude of origin. The children’s living conditions prior to adoption were also recorded, noting whether they had been in institutional care, foster care, or living with their biological families. Hematological parameters, such as hemoglobin (Hb) levels, mean corpuscular volume (MCV), ferritin, and iron levels, were measured to assess the presence and severity of anemia. Additionally, the health assessments included checks for parasitic infections, vitamin D levels, and calcium levels, among other relevant laboratory parameters.

### Statistical methods

Quantitative variables were described by median and interquartile range (IQR) or mean (SD) according to distribution. Normality of the variables has been assessed with Shapiro–Wilk test. Frequencies and percentages were used for qualitative variables. Associations between categorical variables was studied with Pearson’s chi-square test or Fisher’s exact test as appropriate and between quantitative and qualitative variables with Analysis of Variance (ANOVA) with Bonferroni correction. The occurrence of a Hb level inferior to −2SD and that of presenting together Hb Z score < −2SD for and low ferritin have been considered as a binary outcome and studied with multivariable logistic regression models. Multivariable linear regression models have been used to study the correlation of blood laboratory exams with covariates.

All statistical tests were two-sided and the level of statistical significance was set at 0.05. Data have been analyzed with Stata 18 BE (StataCorp. 2023. Stata Statistical Software: Release 18. College Station, TX: StataCorp LLC).

### Ethical approval

The study was approved by the Ethic Committee of Fondazione Policlinico Universitario A. Gemelli IRCCS (protocol 7041, 13/11/2024).

## Results

Nine hundred and sixty-nine patients were enrolled in the study. Demographic characteristics are presented in Table 1.

**Table 1:** Study population

Demographics	Total
	*N* = 969
**Age at arrival, years**	5.2 (2.8–7.5)
**Female sex**	424 (43.8%)
**Skin color**	
**Non black**	590 (60.9%)
**Black**	379 (39.1%)
**Latitude of provenience**	
**North of Polar Arctic Circle**	2 (0.2%)
**Between Polar Arctic Circle and Tropic of Cancer**	332 (34.3%)
**Between Tropic of Cancer and Equator**	421 (43.4%)
**Between Equator and Tropic of Capricorn**	158 (16.3%)
**Between Tropic of Capricorn and Polar Antarctic Circle**	56 (5.8%)
**Season of arrival**	
**Winter**	269 (27.8%)
**Spring**	240 (24.8%)
**Summer**	153 (15.8%)
**Autumn**	307 (31.7%)
**Temporal distance between arrival and sample, months**	0.2 (0.1–0.3)
**Hosting Solution before adoption**	
**Institution**	778 (80.3%)
**House-family**	83 (8.6%)
**Trusted family**	73 (7.5%)
**Missing**	35 (3.6%)
**Height, m**	1.1 (0.9–1.2)
**Height category**	
**Physiologic**	692 (71.4%)
**Pathologic**	178 (18.4%)
**Missing**	99 (10.2%)
**Weight, kg**	18.2 (12.5–25.0)
**Weight category**	
**Physiologic**	663 (68.4%)
**Pathologic**	213 (22.0%)
**Missing**	93 (9.6%)
**BMI, kg/m**^**2**^	16.0 (15.0–17.0)

Demographic data of the population in study. Data are presented as median (IQR) for continuous measures, and n (%) for categorical measures

**Fig. 1:**
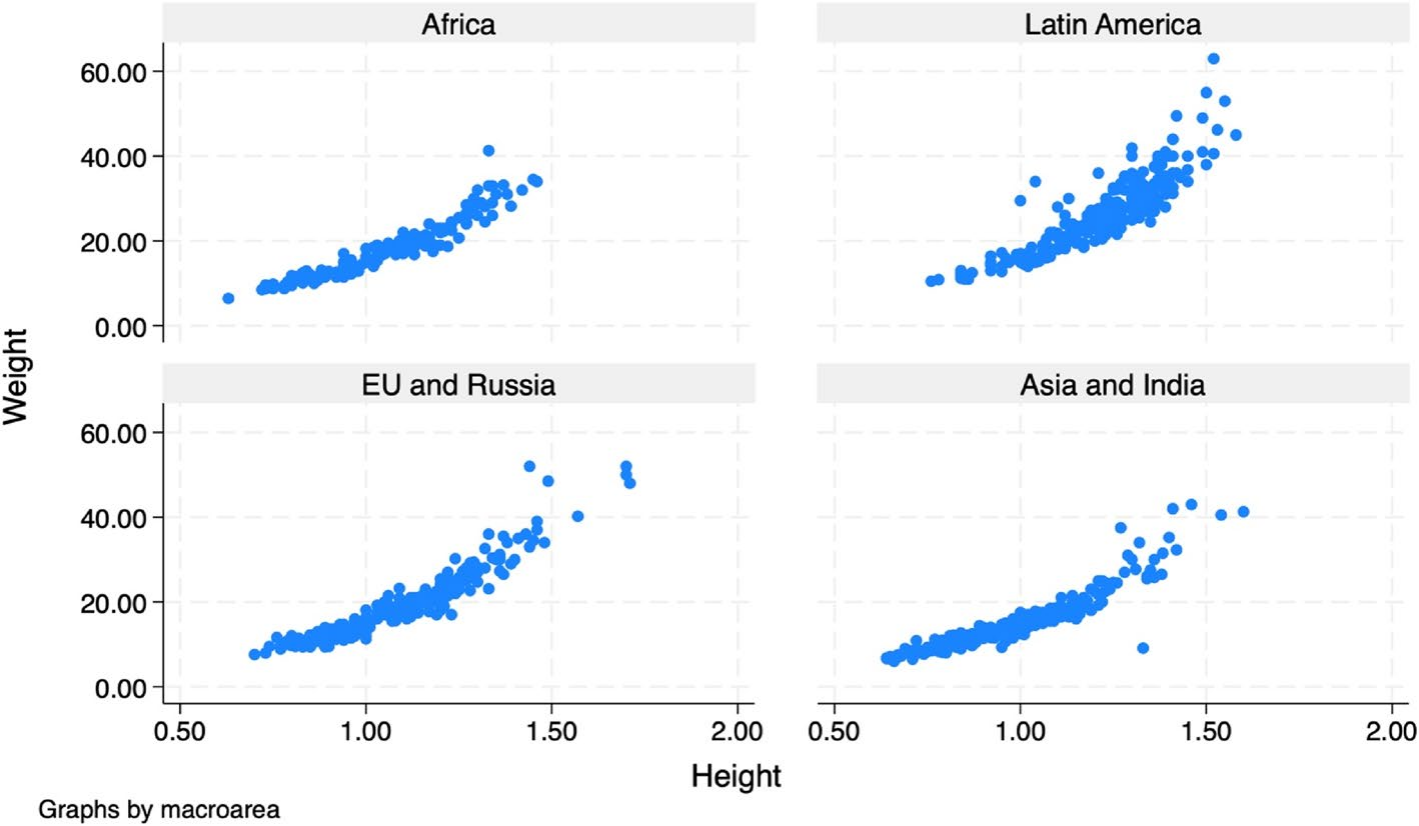
Scatterplot of the relationships between weight and height according to macro-area of origin

Weight and height were significantly lower in children from Asia and India compared to the other macro-areas of origin while they were significantly higher in those from Latin America (*p* < 0.001). The relationship between weight and height for each Macro-area is depicted in Fig. 1.

Laboratory exams for the cohort are presented in Table 1S. Table 2S reports differences in social and demographics as well as laboratory results according to different age groups.

Hemoglobin (Hb) levels were available for 775 patients. Among these, 101 patients (13%) were identified as anemic, defined as having Hb levels below the age-specific reference range.

**Fig. 2:**
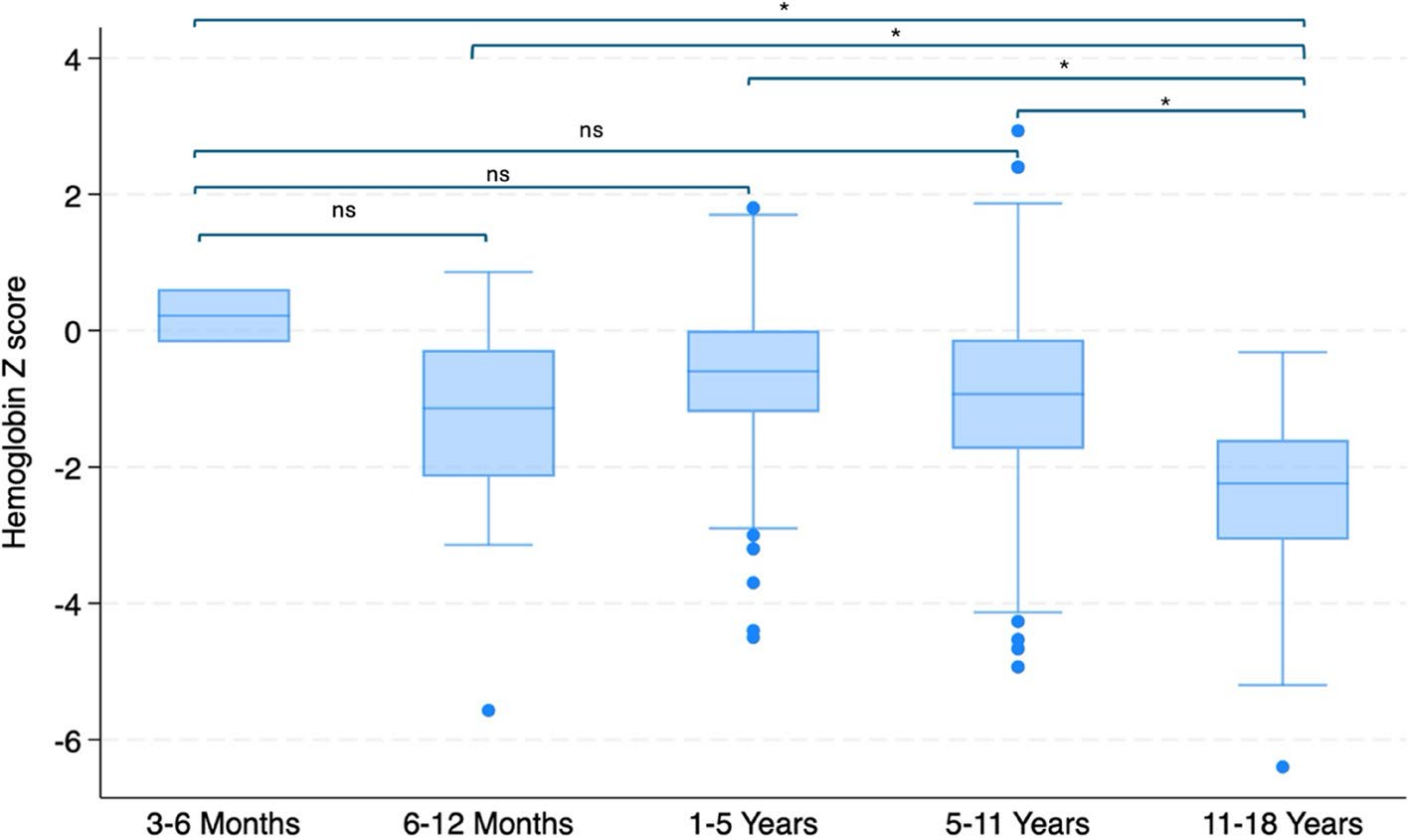
Hemoglobin Z-scores for age category

Statistical analysis highlighted significant correlations between Hb z-scores and certain age categories. Specifically, Hb z-scores were significantly lower in the 11–18 age group compared to all other age groups (*p* < 0.001), and lower in the 5–11 age group compared to the 1–5 age group (Fig. 2).

**Fig. 3:**
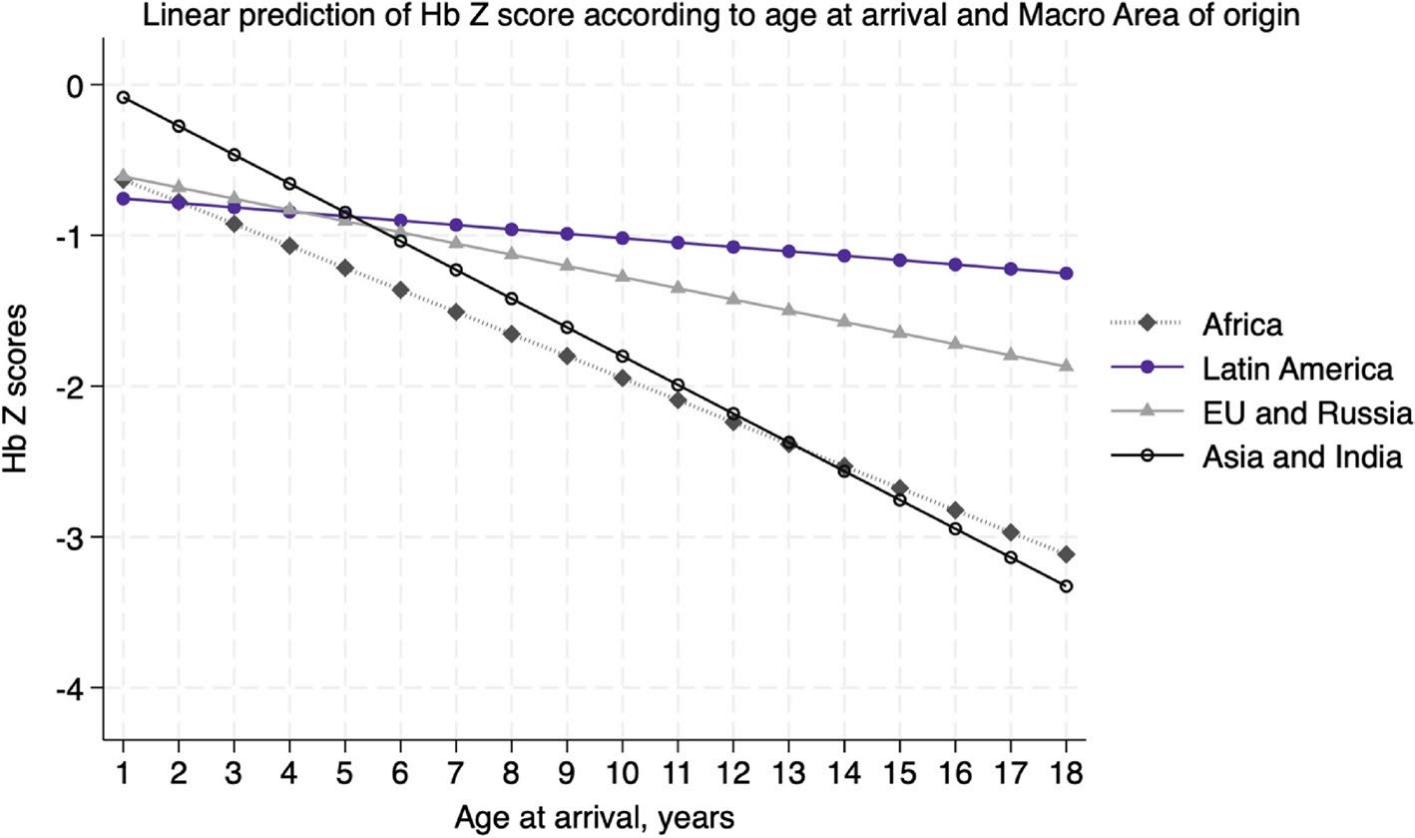
Linear prediction of Hemoglobin Z scores according to age at arrival and Macro Area of origin

In univariate analysis, Hb z-scores were associated with black skin color (Coefficient: −0.47; 95% CI: −0.65 to −0.30; p < 0.001) and the presence of parasites in stool (Coefficient: −0.23; 95% CI: −0.41 to −0.06; p = 0.010). In multivariable analysis, skin color association with Hb Z score was no more significant but there was a relationship with area of origin, age at arrival, MCV, and the relationship with presence of parasites was close to significance (see Table 2, Fig. 3).

**Table 2:** Predictors of Hemoglobin Z scores in adopted children

Hemoglobin Z scores	Coefficient	*P* > t	[95% conf	Interval]
Age at arrival	−0.099	< 0.001	−0.133	−0.064
Mcv	0.092	< 0.001	0.075	0.108
Parassites in stool	−0.168	0.061	−0.344	0.008
Macroarea				
*Latin America*	0.377	0.017	0.068	0.687
*EU and Russia*	0.142	0.412	−0.198	0.481
*Asia and India*	0.339	0.022	0.049	0.629
Color	−0.17	0.158	−0.407	0.066
_cons	−7.369	< 0.001	−8.611	−6.127

Results of multivariable linear regression on Hemoglobin Z scores of the population

Hemoglobin levels were not associated with factors such as Interferon Gamma Releasing Assay positivity, patient BMI, creatinine levels, bilirubin, TSH, FT3, FT4, AST, or ALT.

The mean corpuscular volume (MCV) was associated in univariate analysis with age at arrival, skin color, Macro-area of origin, duration of institutional stay, and several biochemical parameters, including calcium, phosphorus, and iron levels. However, in multivariate analysis that adjusted for ferritin, iron plasma levels, season at the time of sampling, age at arrival, and ALT levels, a clear relationship between these factors and MCV or Hb was not observed, except that, with equal MCV, Hb was higher in Asia compared to Africa (*p* < 0.001) (Table 3S).

**Fig. 4:**
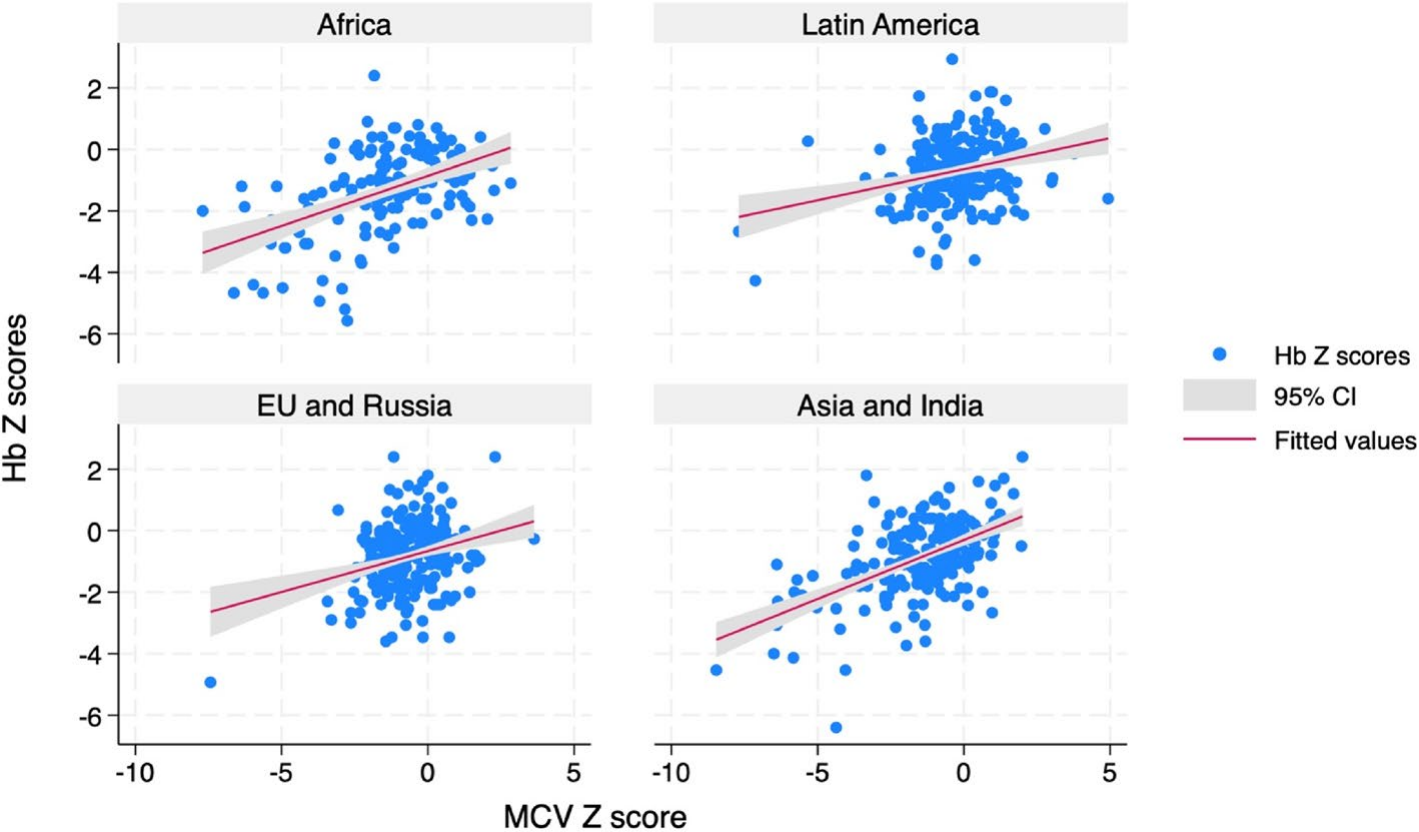
Relationship between Hb Z scores and MCV Z scores in the different MacroAreas of origin

Hemoglobin Z scores were linearly associated with MCV Z scores (Coeff: 0.3; 95% CI: 0.25; 0.35; *p* < 0.001) in univariate analysis. This relationship remained in multivariable analysis including the Macro Area of origin (Fig. 4 and Table 3).

**Table 3:** MCV values associated factors

Hb Z score	Coefficient	*P* > t	[95% conf	Interval]
Mcv Z score	0.30	< 0.001	0.256	0.35
Macroarea				
Latin America	0.28	0.02	0.04	0.53
EU and Russia	0.26	0.03	0.02	0.49
Asia and India	0.47	< 0.001	0.24	0.71
_cons	−0.89	< 0.001	−1.09	−0.69

Results of multivariable linear regression conducted on MCV values. Coefficients of Macro-Areas are compared to Africa as reference

### Anemic patients

Although 101 patients were identified as anemic, only 55 patients had both Hb and MCV values below two standard deviations (Table 4). This subset of patients was predominantly characterized by black skin color and originating from Macro-areas such as Africa and India.

**Table 4:** Anemic and microcitemic children

Characteristic	Total (*N* = 969)	Hb > −2SD (*N* = 914)	Low Hb, Low MCV (*N* = 55)	*p*-value
Female sex	424 (43.8%)	398 (43.5%)	26 (47.3%)	0.59
Black skin	379 (39.1%)	342 (37.4%)	37 (67.3%)	< 0.001
Macroarea—Africa	165 (17.0%)	143 (15.6%)	22 (40.0%)	< 0.001
Macroarea—Latin America	255 (26.3%)	250 (27.4%)	5 (9.1%)	< 0.001
Macroarea—EU and Russia	275 (28.4%)	267 (29.2%)	8 (14.5%)	< 0.001
Macroarea—Asia and India	274 (28.3%)	254 (27.8%)	20 (36.4%)	< 0.001
Age at arrival	5.3 (2.9)	5.3 (2.9)	5.6 (3.4)	0.42
Institute length	3.0 (2.0)	3.0 (2.0)	3.1 (2.8)	0.74
BMI	16.1 (2.6)	16.1 (2.6)	16.2 (2.9)	0.81

Characteristics of patients according to being anemic and microcitemic or not

Ferritin levels were associated with sex, black skin color, abitative solution before adoption (Table 5).

**Table 5:** Ferritine blood levels factors

Ferritine	Coefficient	*P* > t	[95% conf	Interval]
Female sex	3.835	0.017	0.675	6.996
Black color	3.987	0.019	0.65	7.324
Abitative solution				
House-family	1.554	0.578	−3.924	7.031
Trusted family	8.450	0.004	2.757	14.143
Hemoglobin	4.360	< 0.001	2.743	5.977
_cons	−29.297	0.005	−49.734	−8.860

Multivariable linear regression for Ferritine blood levels

Seventy-nine patients had low hemoglobin and low ferritin levels. Their characteristics are presented in the Table 6.

**Table 6:** Low hemoglobin and ferritin

	**Total**	**Normal**	**Low Hb** **Low Ferritin**	***p*-value**
	*N* = 969	*N* = 890	*N* = 79	
**Female**	424 (43.8%)	380 (42.7%)	44 (55.7%)	0.026
**Black skin**	379 (39.1%)	339 (38.1%)	40 (50.6%)	0.029
**Macroarea**				0.029
**Africa**	165 (17.0%)	143 (16.1%)	22 (27.8%)	
**Latin America**	255 (26.3%)	241 (27.1%)	14 (17.7%)	
**EU and Russia**	275 (28.4%)	251 (28.2%)	24 (30.4%)	
**Asia and India**	274 (28.3%)	255 (28.7%)	19 (24.1%)	
**Age at arrival**	5.3 (2.9)	5.2 (3.0)	6.5 (2.2)	< 0.001
**Institute lenght**	3.0 (2.0)	3.0 (2.0)	3.7 (2.6)	0.011

Characteristics of patients according to presenting low Hemoglobin and low Ferritin blood levels. Data are presented as N(%)

Vitamin D levels in the cohort were tested with multivariable linear regression and showed correlations with several factors (Table 7).

**Table 7:** Vitamin D associated factors

Vitamin D	Coefficient	*P* > t	[95% conf	Interval]
Female sex	0.14	0.846	−1.3	1.59
Black skin	−2.2	0.032	−4.21	−0.19
Macroarea				
Latin America	1.16	0.383	−1.45	3.77
EU and Russia	−0.75	0.615	−3.67	2.17
Asia and India	1.54	0.218	−0.91	4
Season				
Spring	4.21	< 0.001	2.29	6.13
Summer	11.51	< 0.001	9.18	13.85
Autumn	4.22	< 0.001	2.37	6.07
Age at arrival	−0.66	< 0.001	−0.95	−0.38
Ca	2.55	0.003	0.85	4.24
Ipth	−0.15	< 0.001	−0.2	−0.1
_cons	0.21	0.982	−17.38	17.8

Factors associated with Vit D levels in multivariable analysis

## Discussion

The findings of this study highlight the significant prevalence of anemia among internationally adopted children. The observed 10.4% prevalence of anemia aligns with previous research, particularly among children adopted from low- and middle-income countries [7].

One of the most compelling findings of this study is the inverse relationship between age at adoption and hemoglobin levels. Children adopted at older ages were more likely to present with lower hemoglobin levels, a result that possibly reflects the prolonged exposure to adverse conditions such as chronic malnutrition, inadequate healthcare, and recurrent infections [8] in their countries of origin [1]. This finding underscores the critical importance of early intervention and comprehensive health assessments for older adopted children, who may have endured longer periods of deprivation before adoption. Addressing anemia in this subgroup requires timely and targeted interventions to mitigate the risk of long-term developmental delays and health complications [3].

The study also revealed significant associations between anemia and geographic origin, particularly Asia and Africa. These findings suggest that both genetic predispositions and environmental factors may play critical roles in the development of anemia. As an example, the Global Burden of Disease showed the prevalence of iron deficiency is particularly represented in Africa and India [9]. Children from Africa, who were shown to have lower hemoglobin levels at the same MCV compared to their counterparts from all other continents, are particularly vulnerable. This finding might lead to hypothesize that also other factors, such as hemoglobinopathies, such as sickle cell disease, along with environmental stressors nutritional deficiencies, are involved in anemia. Hemoglobinopathies are more prevalent in African populations [9]. It also highlights the importance of screening for hemoglobinopathies in adopted children from regions with high prevalence rates, as these conditions may exacerbate the risk of anemia.

Parasitic infections emerged as another significant risk factor for anemia in this population. The association between parasitic infections and lower hemoglobin levels underscores the critical role that chronic infections play in exacerbating anemia [10, 11]. Parasitic infections, which can lead to chronic blood loss, malabsorption of nutrients, and systemic inflammation, are particularly prevalent in children from regions with inadequate sanitation and healthcare infrastructure [12, 13]. Addressing these infections through routine screening and timely treatment is therefore essential for improving the health outcomes of adopted children.

The study’s findings on nutritional deficiencies [14], particularly the high prevalence of vitamin D deficiency and iron deficiency [15], further emphasize the need for comprehensive nutritional assessments. Vitamin D deficiency, which was observed in 78% of the cohort, is a known risk factor for impaired bone health and immune function [16], but also for anemia [17]. Iron deficiency, as indicated by low ferritin levels in 26.9% of the children, is a common cause of anemia that requires prompt intervention to prevent long-term developmental consequences. The association between lower hemoglobin and MCV values with low ferritin and iron levels, particularly among children with black skin, once again points to the heightened vulnerability of African children to deficiency-related anemia. This finding suggests that nutritional interventions, including iron and vitamin D supplementation [18], should be prioritized in the care of these children to address these deficiencies and promote healthy development.

The kind of abitative solution the children had been before adoption also had a role in the overall health status of the cohort. This has been described in other papers, similarly with children from foster care having better outcomes than children who were in orphanage before adoption [7].

Interestingly, the multivariate analysis revealed that some associations observed in the univariate analysis, such as those between hemoglobin levels and black skin color or parasitic infections, were no longer significant when controlling for variables like MacroArea of origin, age at arrival, MCV, and calcium levels. This finding suggests that while these factors may contribute to anemia, their impact is mitigated when considering the broader context of geographic and nutritional factors. For instance, the significance of macroarea in predicting hemoglobin levels highlights the complex interplay between environmental exposures and genetic predispositions in shaping the health outcomes of adopted children.

Moreover, the lack of association between hemoglobin levels and other factors such as quantiferon positivity, BMI, creatinine levels, and thyroid function tests suggests that these parameters may not be central to the anemia observed in this population. However, their inclusion in the analysis is important as it provides a more holistic understanding of the children’s health status and rules out potential confounders.

The finding that anemia in this cohort is predominantly deficiency-related, as evidenced by the association with low ferritin and iron levels, indicates that these conditions are likely to improve with appropriate nutritional and environmental interventions [3]. The fact that these deficiencies are correctable with the adoption of a healthier lifestyle in the new family environment, as supported by literature on post-adoption growth recovery, is encouraging. However, it also highlights the need for ongoing monitoring and support to ensure that these improvements are sustained over time.

The clinical implications of this study are profound. Healthcare providers who care for internationally adopted children should prioritize comprehensive health assessments that include screening for anemia, nutritional deficiencies, and parasitic infections. Given the high prevalence of anemia in this population, early diagnosis and intervention are crucial. Tailored treatment plans should address the specific needs of each child, taking into account factors such as age at adoption, geographic origin, and the presence of underlying conditions like hemoglobinopathies or parasitic infections. Nutritional support, including iron supplementation and vitamin D therapy, should be a key component of care. Additionally, routine follow-up is essential to monitor the effectiveness of interventions and to ensure that the children’s health improves over time.

The findings of this study also have significant public health implications. The high prevalence of anemia and nutritional deficiencies among internationally adopted children highlights the need for public health strategies that address these issues on a broader scale. Public health agencies and adoption services should collaborate to ensure that adopted children receive comprehensive health screenings both before and after adoption. There is also a need for policies that facilitate access to healthcare services for adopted children, including those that support the early identification and treatment of anemia and other health conditions. Furthermore, public health initiatives should focus on educating adoptive families about the potential health challenges their children may face and providing them with the resources and support needed to address these challenges effectively [19].

This study, while comprehensive, is not without its limitations. The retrospective design of the study may limit the ability to establish causal relationships between the observed risk factors and the development of anemia. Additionally, the study relied on medical records from a single clinic, which may limit the generalizability of the findings to other populations or settings. The presence of missing data, particularly concerning iron levels, could also have impacted the accuracy of the results. Furthermore, the study did not account for other potential confounding factors, such as the duration of institutional care before adoption or the socioeconomic status of the adoptive families, which could influence the children’s health outcomes.

Future research should aim to address the limitations of this study by employing a prospective design that allows for the establishment of causal relationships between risk factors and anemia in internationally adopted children. Longitudinal studies that follow children from the time of adoption through their early years in their adoptive homes would provide valuable insights into the long-term health outcomes of this population. Additionally, future research should explore the impact of other potential confounders, such as the duration of pre-adoption institutional care and the role of adoptive family support in mitigating health risks. Further studies should also investigate the effectiveness of different intervention strategies, including nutritional supplementation and treatment for parasitic infections, in improving the health outcomes of adopted children.

## Conclusions

In conclusions, anemia is a prevalent condition among internationally adopted children, influenced by a range of factors including age at adoption, skin color, geographic origin, and the presence of parasitic infections. The findings of this study underscore the critical need for comprehensive health assessments and tailored interventions aimed at addressing the unique health needs of this vulnerable population. By prioritizing early intervention and ongoing monitoring, healthcare providers can help improve the health outcomes and quality of life for internationally adopted children.

## Supplementary Information

Supplementary Material 1.

## Data Availability

Available upon request to the corresponding author.

## Abbreviations

MCV: Mean corpuscola volume
BMI: Body mass index
