# Supplementary material for: Prevalence and risk factors of anemia in internationally adopted children: a cohort study
Source: Ital J Pediatr. 2025 Mar 25;51:99. doi: 10.1186/s13052-025-01944-6 (PMC11934710; doi:10.1186/s13052-025-01944-6)
Supplement: Supplementary file 1 — Supplementary Material 1. [file 13052_2025_1944_MOESM1_ESM.docx]

Supplementary materials

Table 1S

| Blood labs | Total |
| --- | --- |
|  |  |
|  |  |
|  | N=969 |
|  |  |
| Vitamin D | 19.8 (14.0-26.4) |
|  |  |
| Vitamin D category |  |
| Physiologic | 145 (15.0%) |
| Pathologic | 756 (78.0%) |
| Missing | 68 (7.0%) |
|  |  |
| Paratohormone | 29.9 (22.5-38.6) |
|  |  |
| Pth category |  |
| Physiologic | 852 (87.9%) |
| Pathologic | 38 (3.9%) |
| Missing | 79 (8.2%) |
|  |  |
| Calcium | 9.9 (9.6-10.2) |
|  |  |
| Calcium category |  |
| Physiologic | 732 (75.5%) |
| Pathologic | 122 (12.6%) |
| Missing | 115 (11.9%) |
|  |  |
| Phosphate | 4.8 (4.5-5.2) |
|  |  |
| Phosphate cat |  |
| Physiologic | 813 (83.9%) |
| Pathologic | 20 (2.1%) |
| Missing | 136 (14.0%) |
|  |  |
| Magnesium | 2.2 (2.1-2.3) |
|  |  |
| Magnesium category |  |
| Physiologic | 602 (62.1%) |
| Pathologic | 43 (4.4%) |
| Missing | 324 (33.4%) |
|  |  |
| Alkaline phosphatase | 281.0 (224.0-483.0) |
|  |  |
| Alkaline phosphatase category |  |
| < 300 | 509 (52.5%) |
| > 300 | 404 (41.7%) |
| Missing | 56 (5.8%) |
|  |  |
| Stool parasite examination |  |
| Negative | 349 (36.0%) |
| Positive | 439 (45.3%) |
| Missing | 181 (18.7%) |
|  |  |
| Quantiferon (positive) | 44 (5.2%) |
|  |  |
| Mitogen | 0.0 (0.0-0.0) |
|  |  |
| mitogen | 10.1 (9.1-10.1) |
|  |  |
| Hemoglobin | 12.5 (11.9-13.1) |
|  |  |
| Hemoglobin category |  |
| Physiologic | 674 (69.6%) |
| Pathologic | 101 (10.4%) |
| Missing | 194 (20.0%) |
|  |  |
| Mcv | 77.4 (74.2-80.1) |
|  |  |
| Mcv category |  |
|  | 1 (0.1%) |
| Physiologic | 208 (21.5%) |
| Pathologic | 576 (59.4%) |
| Missing | 184 (19.0%) |
|  |  |
| Plt | 310.0 (268.0-367.0) |
|  |  |
| Plt category |  |
| Physiologic | 724 (74.7%) |
| Pathologic | 44 (4.5%) |
| Missing | 201 (20.7%) |
|  |  |
| Wbc | 7.8 (6.2-9.3) |
|  |  |
| Wbc cat. |  |
| Physiologic | 599 (61.8%) |
| Pathologic | 171 (17.6%) |
| Missing | 199 (20.5%) |
|  |  |
| Neutr | 3.0 (2.2-3.9) |
|  |  |
| Neutr cat. |  |
| Physiologic | 654 (67.5%) |
| Pathologic | 113 (11.7%) |
| Missing | 202 (20.8%) |
|  |  |
| Lymph | 3.5 (2.7-4.6) |
|  |  |
| Lymph cat |  |
| Physiologic | 628 (64.8%) |
| Pathologic | 139 (14.3%) |
| Missing | 202 (20.8%) |
|  |  |
| eosin | 0.2 (0.1-0.4) |
|  |  |
| eosin_cat |  |
| Physiologic | 634 (65.4%) |
| Pathologic | 136 (14.0%) |
| Missing | 199 (20.5%) |
|  |  |
| Ferritine | 24.0 (16.0-36.0) |
|  |  |
| ferrit_cat |  |
| Physiologic | 520 (53.7%) |
| Pathologic | 261 (26.9%) |
| Missing | 188 (19.4%) |
|  |  |
| Iron | 75.0 (57.0-99.0) |
|  |  |
| Iron category |  |
| Physiologic | 347 (35.8%) |
| Pathologic | 165 (17.0%) |
| Missing | 457 (47.2%) |
|  |  |
|  |  |
| crea | 0.4 (0.3-0.5) |
|  |  |
| crea_cat |  |
| 0 | 781 (80.6%) |
| Missing | 188 (19.4%) |
|  |  |
| Alt | 17.0 (14.0-21.0) |
|  |  |
| Alt cat |  |
| Physiologic | 751 (77.5%) |
| Pathologic | 16 (1.7%) |
| Missing | 202 (20.8%) |
|  |  |
| Ast | 34.0 (28.0-40.0) |
|  |  |
| Ast cat |  |
| Physiologic | 105 (10.8%) |
| Pathologic | 18 (1.9%) |
| Missing | 846 (87.3%) |
|  |  |
| Tsh | 2.5 (1.6-3.4) |
|  |  |
| Tsh cat |  |
| Physiologic | 299 (30.9%) |
| Pathologic | 15 (1.5%) |
| Missing | 655 (67.6%) |
|  |  |
| Ft3 | 4.3 (3.8-4.8) |
|  |  |
| ft3 category |  |
| Physiologic | 182 (18.8%) |
| Pathologic | 35 (3.6%) |
| Missing | 752 (77.6%) |
|  |  |
| Ft4 | 12.1 (11.2-13.2) |
|  |  |
| ft4 category |  |
| Physiologic | 239 (24.7%) |
| Pathologic | 4 (0.4%) |
| Missing | 726 (74.9%) |

Table 1S: Blood laboratory exams in the cohort. Data are expressed as N(%) or median (IQR).

Table 2S

| Age categories | Total | 3-6  Months | 6-12 Months | 1-5  Years | 5-11  Years | 11-18  Years | p-value |
| --- | --- | --- | --- | --- | --- | --- | --- |
|  |  |  |  |  |  |  |  |
|  |  |  |  |  |  |  |  |
|  | N=969 | N=3 | N=32 | N=400 | N=500 | N=34 |  |
|  |  |  |  |  |  |  |  |
| Latitude |  |  |  |  |  |  | <0.001 |
| 1 | 2 (0.2%) | 0 (0.0%) | 0 (0.0%) | 2 (0.5%) | 0 (0.0%) | 0 (0.0%) |  |
| 2 | 332 (34.3%) | 1 (33.3%) | 3 (9.4%) | 139 (34.8%) | 178 (35.6%) | 11 (32.4%) |  |
| 3 | 421 (43.4%) | 0 (0.0%) | 29 (90.6%) | 211 (52.8%) | 165 (33.0%) | 16 (47.1%) |  |
| 4 | 158 (16.3%) | 2 (66.7%) | 0 (0.0%) | 44 (11.0%) | 107 (21.4%) | 5 (14.7%) |  |
| 5 | 56 (5.8%) | 0 (0.0%) | 0 (0.0%) | 4 (1.0%) | 50 (10.0%) | 2 (5.9%) |  |
|  |  |  |  |  |  |  |  |
| Black skin | 379 (39.1%) | 2 (66.7%) | 6 (18.8%) | 159 (39.8%) | 199 (39.8%) | 13 (38.2%) | 0.15 |
| Female sex | 424 (43.8%) | 1 (33.3%) | 15 (46.9%) | 166 (41.5%) | 222 (44.4%) | 20 (58.8%) | 0.37 |
|  |  |  |  |  |  |  |  |
| Season |  |  |  |  |  |  | 0.32 |
| *Winter* | 269 (27.8%) | 0 (0.0%) | 9 (28.1%) | 113 (28.2%) | 136 (27.2%) | 11 (32.4%) |  |
| *Spring* | 240 (24.8%) | 1 (33.3%) | 9 (28.1%) | 100 (25.0%) | 122 (24.4%) | 8 (23.5%) |  |
| *Summer* | 153 (15.8%) | 1 (33.3%) | 2 (6.2%) | 72 (18.0%) | 69 (13.8%) | 9 (26.5%) |  |
| *Autumn* | 307 (31.7%) | 1 (33.3%) | 12 (37.5%) | 115 (28.7%) | 173 (34.6%) | 6 (17.6%) |  |
|  |  |  |  |  |  |  |  |
| Institute lenght (months) | 3.0 (2.0) | 5.9(2) | 0.6 (0.1) | 2.2 (1.1) | 3.7 (2.2) | 5.5 (3.2) | <0.001 |
|  |  |  |  |  |  |  |  |
|  |  |  |  |  |  |  |  |
| Solution |  |  |  |  |  |  | 0.001 |
| *Institution* | 778 (80.3%) | 2 (66.7%) | 30 (93.8%) | 341 (85.2%) | 383 (76.6%) | 22 (64.7%) |  |
| *Foster Family* | 83 (8.6%) | 0 (0.0%) | 1 (3.1%) | 23 (5.8%) | 55 (11.0%) | 4 (11.8%) |  |
| *Trusted Family* | 73 (7.5%) | 1 (33.3%) | 0 (0.0%) | 18 (4.5%) | 48 (9.6%) | 6 (17.6%) |  |
| *Missing* | 35 (3.6%) | 0 (0.0%) | 1 (3.1%) | 18 (4.5%) | 14 (2.8%) | 2 (5.9%) |  |
| Height | 1.2 (3.1) | 1.3 (.) | 0.7 (0.0) | 1.2 (5.0) | 1.2 (0.1) | 1.4 (0.2) | 0.94 |
| Height category |  |  |  |  |  |  | <0.001 |
| 0 | 692 (71.4%) | 1 (33.3%) | 17 (53.1%) | 248 (62.0%) | 399 (79.8%) | 27 (79.4%) |  |
| 1 | 178 (18.4%) | 0 (0.0%) | 7 (21.9%) | 98 (24.5%) | 69 (13.8%) | 4 (11.8%) |  |
| Missing | 99 (10.2%) | 2 (66.7%) | 8 (25.0%) | 54 (13.5%) | 32 (6.4%) | 3 (8.8%) |  |
| Weight | 19.8 (8.9) | 20.1 (17.7) | 7.7 (1.1) | 13.1 (3.7) | 24.3 (6.8) | 38.5 (10.7) | <0.001 |
| Weight category |  |  |  |  |  |  | <0.001 |
| 0 | 663 (68.4%) | 1 (33.3%) | 14 (43.8%) | 231 (57.8%) | 390 (78.0%) | 27 (79.4%) |  |
| 1 | 213 (22.0%) | 1 (33.3%) | 11 (34.4%) | 119 (29.8%) | 78 (15.6%) | 4 (11.8%) |  |
| Missing | 93 (9.6%) | 1 (33.3%) | 7 (21.9%) | 50 (12.5%) | 32 (6.4%) | 3 (8.8%) |  |
| Bmi | 16.1 (2.6) | 19.0 (.) | 17.0 (.) | 15.4 (1.8) | 16.3 (2.8) | 18.1 (3.1) | <0.001 |
| Bmi category |  |  |  |  |  |  | <0.001 |
| 0 | 549 (56.7%) | 0 (0.0%) | 1 (3.1%) | 202 (50.5%) | 328 (65.6%) | 18 (52.9%) |  |
| 1 | 94 (9.7%) | 0 (0.0%) | 0 (0.0%) | 51 (12.8%) | 39 (7.8%) | 4 (11.8%) |  |
| 2 | 140 (14.4%) | 1 (33.3%) | 0 (0.0%) | 45 (11.2%) | 86 (17.2%) | 8 (23.5%) |  |
| Missing | 186 (19.2%) | 2 (66.7%) | 31 (96.9%) | 102 (25.5%) | 47 (9.4%) | 4 (11.8%) |  |
|  |  |  |  |  |  |  |  |
| Vit D | 21.1 (11.8) | 17.7 (1.7) | 39.0 (19.2) | 22.8 (12.2) | 19.0 (9.8) | 18.1 (9.7) | <0.001 |
|  |  |  |  |  |  |  |  |
| ipth | 32.3 (15.5) | 36.0 (16.5) | 27.6 (9.8) | 30.0 (13.5) | 33.8 (16.8) | 40.0 (16.0) | <0.001 |
|  |  |  |  |  |  |  |  |
| Pth cat |  |  |  |  |  |  | 0.15 |
| 0 | 852 (87.9%) | 2 (66.7%) | 27 (84.4%) | 343 (85.8%) | 450 (90.0%) | 30 (88.2%) |  |
| 1 | 38 (3.9%) | 0 (0.0%) | 0 (0.0%) | 16 (4.0%) | 20 (4.0%) | 2 (5.9%) |  |
| Missing | 79 (8.2%) | 1 (33.3%) | 5 (15.6%) | 41 (10.2%) | 30 (6.0%) | 2 (5.9%) |  |
|  |  |  |  |  |  |  |  |
| Ca++ | 9.9 (0.5) | 9.9 (0.4) | 10.4 (0.4) | 10.0 (0.5) | 9.9 (0.4) | 9.8 (0.5) | <0.001 |
|  |  |  |  |  |  |  |  |
| Ca cat |  |  |  |  |  |  | <0.001 |
| 0 | 732 (75.5%) | 2 (66.7%) | 14 (43.8%) | 286 (71.5%) | 402 (80.4%) | 28 (82.4%) |  |
| 1 | 122 (12.6%) | 0 (0.0%) | 11 (34.4%) | 61 (15.2%) | 47 (9.4%) | 3 (8.8%) |  |
| Missing | 115 (11.9%) | 1 (33.3%) | 7 (21.9%) | 53 (13.2%) | 51 (10.2%) | 3 (8.8%) |  |
|  |  |  |  |  |  |  |  |
| Phosphate | 4.9 (0.6) | 4.3 (1.8) | 5.5 (0.7) | 5.0 (0.6) | 4.7 (0.5) | 4.6 (0.7) | <0.001 |
|  |  |  |  |  |  |  |  |
| P cat |  |  |  |  |  |  | 0.025 |
| 0 | 813 (83.9%) | 2 (66.7%) | 21 (65.6%) | 333 (83.2%) | 427 (85.4%) | 30 (88.2%) |  |
| 1 | 20 (2.1%) | 0 (0.0%) | 1 (3.1%) | 4 (1.0%) | 15 (3.0%) | 0 (0.0%) |  |
| Missing | 136 (14.0%) | 1 (33.3%) | 10 (31.2%) | 63 (15.8%) | 58 (11.6%) | 4 (11.8%) |  |
|  |  |  |  |  |  |  |  |
| Mg | 2.2 (0.2) | 2.2 (0.1) | 2.3 (0.1) | 2.2 (0.2) | 2.1 (0.2) | 2.1 (0.1) | <0.001 |
|  |  |  |  |  |  |  |  |
| Mg cat |  |  |  |  |  |  | <0.001 |
| 0 | 602 (62.1%) | 2 (66.7%) | 8 (25.0%) | 238 (59.5%) | 336 (67.2%) | 18 (52.9%) |  |
| 1 | 43 (4.4%) | 0 (0.0%) | 2 (6.2%) | 25 (6.2%) | 16 (3.2%) | 0 (0.0%) |  |
| Missing | 324 (33.4%) | 1 (33.3%) | 22 (68.8%) | 137 (34.2%) | 148 (29.6%) | 16 (47.1%) |  |
|  |  |  |  |  |  |  |  |
| Alk phosphatase | 389.0  (298.5) | 229.5  (14.8) | 490.8 (235.2) | 382.9 (354.4) | 384.4 (248.6) | 437.7 (320.2) | 0.26 |
|  |  |  |  |  |  |  |  |
| Alk p cat |  |  |  |  |  |  | 0.011 |
| < 300 | 509 (52.5%) | 2 (66.7%) | 9 (28.1%) | 218 (54.5%) | 265 (53.0%) | 15 (44.1%) |  |
| > 300 | 404 (41.7%) | 0 (0.0%) | 22 (68.8%) | 154 (38.5%) | 210 (42.0%) | 18 (52.9%) |  |
| Missing | 56 (5.8%) | 1 (33.3%) | 1 (3.1%) | 28 (7.0%) | 25 (5.0%) | 1 (2.9%) |  |
|  |  |  |  |  |  |  |  |
| Parass stool |  |  |  |  |  |  | <0.001 |
| Negative | 349 (36.0%) | 2 (66.7%) | 19 (59.4%) | 176 (44.0%) | 147 (29.4%) | 5 (14.7%) |  |
| Positive | 439 (45.3%) | 0 (0.0%) | 3 (9.4%) | 140 (35.0%) | 275 (55.0%) | 21 (61.8%) |  |
| Missing | 181 (18.7%) | 1 (33.3%) | 10 (31.2%) | 84 (21.0%) | 78 (15.6%) | 8 (23.5%) |  |
|  |  |  |  |  |  |  |  |
| Qtf pos |  |  |  |  |  |  | 0.03 |
| 0 | 799 (82.5%) | 2 (66.7%) | 23 (71.9%) | 331 (82.8%) | 419 (83.8%) | 24 (70.6%) |  |
| 1 | 44 (4.5%) | 0 (0.0%) | 2 (6.2%) | 10 (2.5%) | 28 (5.6%) | 4 (11.8%) |  |
| Missing | 126 (13.0%) | 1 (33.3%) | 7 (21.9%) | 59 (14.8%) | 53 (10.6%) | 6 (17.6%) |  |
|  |  |  |  |  |  |  |  |
| Mtb ag | 0.2 (0.9) | 0.0 (0.0) | 0.1 (0.1) | 0.1 (0.8) | 0.2 (0.8) | 1.0 (2.7) | 0.004 |
|  |  |  |  |  |  |  |  |
| Mitogen | 9.0 (2.1) | 9.0 (1.6) | 9.4 (1.3) | 8.9 (2.2) | 9.0 (2.2) | 9.5 (1.5) | 0.87 |
|  |  |  |  |  |  |  |  |
| Hb | 12.5 (1.0) | 12.1 (0.6) | 11.8 (1.0) | 12.2 (1.0) | 12.7 (1.0) | 13.0 (0.9) | <0.001 |
|  |  |  |  |  |  |  |  |
| Hb z | -0.9 (1.2) | 0.2 (0.6) | -1.3 (1.5) | -0.7 (1.0) | -1.0 (1.3) | -2.5 (1.3) | <0.001 |
|  |  |  |  |  |  |  |  |
| Mcv | 76.7 (5.7) | 75.2 (5.7) | 69.4 (7.4) | 75.0 (5.1) | 78.3 (5.2) | 79.9 (6.1) | <0.001 |
|  |  |  |  |  |  |  |  |
| Plt | 322.1 (77.4) | 266.0 (42.4) | 353.6 (94.3) | 332.4 (81.9) | 315.0 (72.1) | 289.8 (65.9) | 0.001 |
|  |  |  |  |  |  |  |  |
| Wbc | 8.1 (2.6) | 6.4 (0.3) | 11.0 (2.2) | 9.0 (2.7) | 7.3 (2.1) | 6.7 (2.1) | <0.001 |
|  |  |  |  |  |  |  |  |
| Eosinophils | 0.3 (0.4) | 0.3 (0.2) | 0.3 (0.2) | 0.3 (0.3) | 0.4 (0.5) | 0.4 (0.5) | 0.15 |
|  |  |  |  |  |  |  |  |
| Ferritine | 28.9 (21.3) | 25.0 (0.0) | 27.8 (14.5) | 26.0 (20.6) | 31.3 (22.0) | 25.3 (18.1) | 0.018 |
|  |  |  |  |  |  |  |  |
| Iron | 77.6 (30.6) | 135.0 (.) | 74.9 (12.6) | 74.8 (32.2) | 79.3 (30.1) | 82.1 (29.1) | 0.16 |
|  |  |  |  |  |  |  |  |
|  |  |  |  |  |  |  |  |
| Creatinine | 0.4 (0.1) | 0.4 (0.1) | 0.4 (0.2) | 0.3 (0.1) | 0.4 (0.1) | 0.6 (0.1) | <0.001 |
|  |  |  |  |  |  |  |  |
| Alt | 20.5 (23.9) | 25.5 (7.8) | 44.2 (65.1) | 22.9 (31.3) | 17.6 (6.8) | 15.9 (7.8) | <0.001 |
|  |  |  |  |  |  |  |  |
| Ast | 36.0 (15.5) |  | 67.9 (48.9) | 37.8 (8.0) | 31.6 (6.6) | 27.1 (5.4) | <0.001 |
|  |  |  |  |  |  |  |  |
| Tsh | 2.8 (1.5) |  | 3.3 (0.8) | 3.1 (1.5) | 2.5 (1.3) | 3.4 (2.9) | 0.002 |
|  |  |  |  |  |  |  |  |
| ft3 | 4.3 (0.7) |  | 4.5 (0.5) | 4.5 (0.7) | 4.2 (0.7) | 4.0 (0.4) | 0.005 |
|  |  |  |  |  |  |  |  |
| ft4 | 12.3 (1.7) |  | 14.2 (0.4) | 12.7 (1.8) | 12.0 (1.4) | 11.5 (1.8) | <0.001 |
|  |  |  |  |  |  |  |  |
|  |  |  |  |  |  |  |  |

Table 2S: Socio-demographic and laboratory exams of the cohort according to age category. Data are expressed as N(%) or median (IQR).

Table 3S

| Mcv | Coefficient | P>t | [95% conf. | interval] |
| --- | --- | --- | --- | --- |
|  |  |  |  |  |
| Ferritine | 0.036 | 0.01 | 0.008 | 0.064 |
| Iron | 0.045 | <0.001 | 0.0297 | 0.061 |
| Season | -0.515 | 0.013 | -0.924 | -0.107 |
| Age arrival | 0.657 | <0.001 | 0.492 | 0.823 |
| Alt | -0.047 | 0.009 | -0.083 | -0.011 |

Table 3S: results of multivariable linear regression for MCV.
